# Knock out of the BASIGIN/CD147 chaperone of lactate/H+ symporters disproves its pro-tumour action *via* extracellular matrix metalloproteases (MMPs) induction

**DOI:** 10.18632/oncotarget.4323

**Published:** 2015-05-29

**Authors:** Ibtissam Marchiq, Jean Albrengues, Sara Granja, Cédric Gaggioli, Jacques Pouysségur, Marie-Pierre Simon

**Affiliations:** ^1^ INSERM, CNRS, Institute for Research on Cancer and Aging, Nice (IRCAN), University of Nice Sophia Antipolis, Centre Antoine Lacassagne, Nice, France; ^2^ INSERM, CNRS, Institute for Research on Cancer and Aging, Nice (IRCAN), University of Nice Sophia Antipolis, Medical School, Nice, France; ^3^ Life and Health Sciences Research Institute (ICVS), School of Health Sciences, University of Minho, Campus of Gualtar, Braga, Portugal; ^4^ ICVS/3B's-PT Government Associate Laboratory, Braga/Guimarães, Portugal; ^5^ Centre Scientifique de Monaco (CSM), Quai Antoine Ier MC, France

**Keywords:** cancer biology, membrane transport, MCT, lactic acid, glycolysis

## Abstract

BASIGIN/CD147/EMMPRIN is a multifunctional transmembrane glycoprotein strongly expressed in tumours. BASIGIN controls tumour metabolism, particularly glycolysis by facilitating lactic acid export through the two monocarboxylate transporters MCT1 and hypoxia-inducible MCT4. However, before being recognized as a co-carrier of MCTs, BASIGIN was described as an inducer of extracellular matrix metalloproteases (MMPs). Early on, a model emerged in which, tumour cells use the extracellular domain of BASIGIN to recognize and stimulate neighbouring fibroblasts to produce MMPs. However, this model has remained hypothetical since a direct link between BASIGIN and MMPs production has not yet been clearly established. To validate the BASIGIN/MMP hypothesis, we developed BASIGIN knockouts in three human tumour cell lines derived from glioma, colon, and lung adenocarcinoma. By using co-culture experiments of either human or mouse fibroblasts and tumour cell lines we showed, contrary to what has been abundantly published, that the disruption of BASIGIN in tumour cells and in MEFs has no action on the production of MMPs. Our findings do not support the notion that the pro-tumoural action of BASIGIN is mediated *via* induction of MMPs. Therefore, we propose that to date, the strongest pro-tumoural action of BASIGIN is mediated through the control of fermentative glycolysis.

## INTRODUCTION

BASIGIN/CD147 (BSG) is a highly glycosylated transmembrane protein belonging to the immunoglobulins (Ig) super family. The main isoform described (BSG-2) is composed of two ( Ig-like) extracellular domains, a single membrane spanning segment followed by an intracellular domain. BASIGIN is described as a multifunctional protein that could work in conjunction with several partners and therefore be involved in many cellular functions. At the physiological level, it plays a role in embryonic development (spermatogenesis, vision and smell), adhesion and the inflammatory processes [1–3]. Furthermore, BASIGIN plays an important role in regulating the energy metabolism of cells through its association with the MonoCarboxylate Transporters (bidirectional lactate/H+ symporters), MCT1 and MCT4, [4–8]. Moreover, BASIGIN is involved in several diseases including inflammatory asthma, arthritis, and auto-immune pathologies such as HIV-1 infection, corona virus and hepatitis B and C [2]. Interestingly, BASIGIN was recently identified as the entry gate for Plasmodium Falciparum PfRh5, leading to the spread of malaria [2, 9], and for the meningococcal pilus component PilE and PilV [10]. BASIGIN is a highly conserved protein in evolution, is expressed in all cell types of metazoans, and is overexpressed in conjunction with MCTs in a variety of rapidly growing tumours. Furthermore, this BASIGIN/MCTs overexpression in cancers is directly correlated with a poor prognosis for survival [2, 3, 11, 12]. BASIGIN is involved in cancer at two main levels. First, we and others have demonstrated that BASIGIN acts in cancer progression by controlling the energy metabolism of glycolytic tumours *via* its tight association with lactic acid carriers MCT1 and MCT4 [13–16]. Nevertheless, before being recognized as a chaperone of MCTs, BASIGIN (alternatively named EMMPRIN for Extracellular Matrix MetalloPRotease INducer) was reported to increase tumour growth and metastasis *via* its capacity to induce the expression of extra cellular matrix metalloproteases (MMPs) and to modify the tumour microenvironment [17–19]. This invasive capacity was also associated with the HIF1-mediated induction of VEGF and its receptor VEGFR2 [20–22]. However the mechanism by which BASIGIN mediates these actions is still unclear. A large number of observations suggest that BASIGIN works through its first extracellular Ig-like domain. Tumour cells would secrete molecules of soluble BASIGIN into the extracellular medium that are capable to induce the production of MMPs after homotypic interaction with surrounding fibroblasts [23]. Thus, BASIGIN could be directly involved in the regulation of tumour growth, invasion and metastasis by acting on both stromal and tumour cells, through the induction of angiogenic factors and proteases. During tumour progression, many interactions take place between cancer cells and the stroma, which constitutes their immediate environment. The stroma is mostly composed of capillaries, immune cells, fibroblasts and the extracellular matrix. Fibroblasts are the most abundant cells of the stroma, and a key cellular component of tumours. Around cancer cells, most of the fibroblasts acquire an activated status, and are known as carcinoma associated fibroblasts (CAF). The presence of CAFs is often associated with bad clinical prognosis, and a number of recent studies indicate that CAFs could play an important role in all steps of tumour progression, from initiation until metastasis [24–26]. Therefore, the notion that BASIGIN could serve as an inducer of MMPs is an attractive and interesting hypothesis in the context of tumour microenvironment and metastasis.

However, the model placing BASIGIN at the centre of MMPs induction remains hypothetical. Links established between BASIGIN, MMPs and invasion are often indirect and the results obtained based on siRNA knockdown or forced expression and incubation with recombinant BASIGIN, are unconvincing. To gain insight into the role of BASIGIN in the tumour microenvironment, we genetically disrupted BASIGIN in three human tumour cell lines derived from glioma, colon, and lung adenocarcinoma using zinc finger nuclease (ZFN) technology. The effect of BASIGIN knockout on tumour microenvironment regulation, and in particular on fibroblasts activation, was tested *in vitro* on co-cultures of tumour cells lines (wild type *versus BSG*-null cells) with human or mouse embryonic fibroblasts. In these models, we focused on the expression of MMPs usually described to be associated with tumour invasion, and correlated with the expression of BASIGIN. Among these MMPs, we observed collagenases MMP1 and MMP13, stromelysins MMP3 and MMP11, the membrane type (MT) 1-MMP, MMP14, and finally the most described gelatinases A and B MMP2 and MMP9 [27, 28]. However, in contradiction with the abundant literature, our findings do not support the model of BASIGIN/EMMPRIN as a key player in tumour invasion *via* MMP induction.

## RESULTS

### ZFN-mediated gene knockout of BSG isoform 2

**Figure 1:**
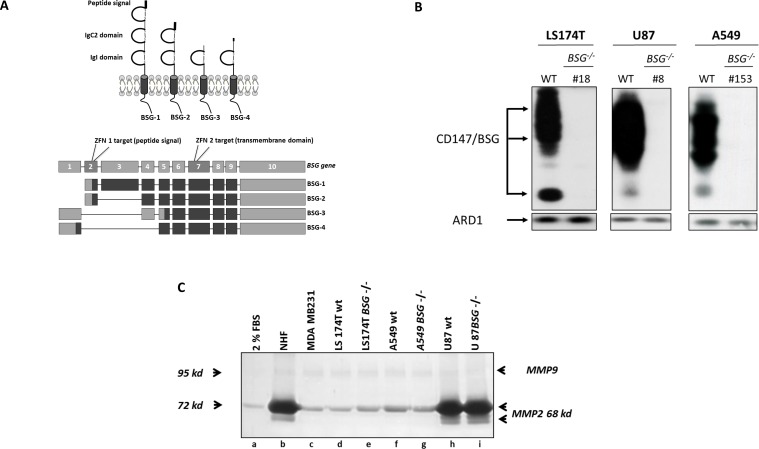
Zinc Finger Nucleases Knock out of BASIGIN/CD147 gene in tumour cells **A.** Schematic representation of the four isoforms of human BASIGIN (BSG-1, BSG-2, BSG-3 and BSG-4), and mapping of the human *BASIGIN* gene sequence (exons/introns) with the four mRNA sequences for human BASIGIN spliced isoforms adapted from [29]. Two ZFN were used to generate *BSG*^−/−^ cell lines; ZFN1 targeted the exon 2 encoding peptide signal and common to BSG-1 and BSG-2, while ZFN2 was designed to target the exon 7, encoding a part of the transmembrane domain and common to all the isoforms. **B.** Western blot analysis. LS174T, U87 and A549 wt cells and *BSG*^−/−^ clones were grown in normoxia during 48h and lysed. BASIGIN expression was analysed by immunoblotting. ARD1 was used as loading control. **C.** Zymogel analysis of Conditioned Media (CM) from wt *vs BSG*^−/−^ tumour cells lines. Cells were grown for 48h in 2% FBS media (lane a). Conditioned media were harvested, and MMP2/MMP9 activities were analysed by loading ECM samples in a 10% polyacrylamide zymogel.

We knocked out the *BASIGIN* gene using ZFN technology in LS174T, U87 and A549 cells. Several clones were obtained for all cell lines (Table 1). Among them, we selected LS174T *BSG*^−/−^ cl.18, U87 *BSG*^−/−^ cl.8 and cl.39 and A549 *BSG*^−/−^ cl.153. BSG-2 isoform knockout (Figure 1A) caused the total loss of the two forms of the protein (highly glycosylated and low non mature molecular weight) in the three cell lines (Figure 1B). Clonogenicity and growth rate analysis (data not shown) of LS174T, U87, and A549 wt *versus BSG*^−/−^ cells showed that BASIGIN disruption had moderate if any effect on clonal growth [16]. This result was surprising in light of shRNA effects previously reported in the literature [13, 14]. Thus we isolated independent BSG-null clones by targeting exon 7 of the *BSG* gene (Figure 1A). This exon contains the site previously chosen for shRNA and is common to all BASIGIN spliced isoforms [29]. The growth phenotype, metabolic profile and MCT expression of these new LS174 BSG-null clones were identical to those obtained by targeting *BSG* exon 2 (data not shown). This finding confirms that in tumour cells, BSG-2 is the most abundant if not the only expressed spliced form of BASIGIN [1–3].

**Table 1:** Primary cell cultures and cell lines used in the study

NHF: Normal Human Fibroblasts
MEF wt: Mouse Embryonic Fibroblast MEF BSG^−/−^
LS174T wt: Human colon carcinoma cell line LS174T BSG^−/−^: clone 18
U 87 wt: Human glioblastoma cell line U 87 BSG^−/−^: clone 8 and 39
A549 wt: Lung carcinoma cell line A549 BSG^−/−^: clone 153

### Human tumour cells express varying levels of MMP2 and MMP9 in extracellular medium

**Figure 2:**
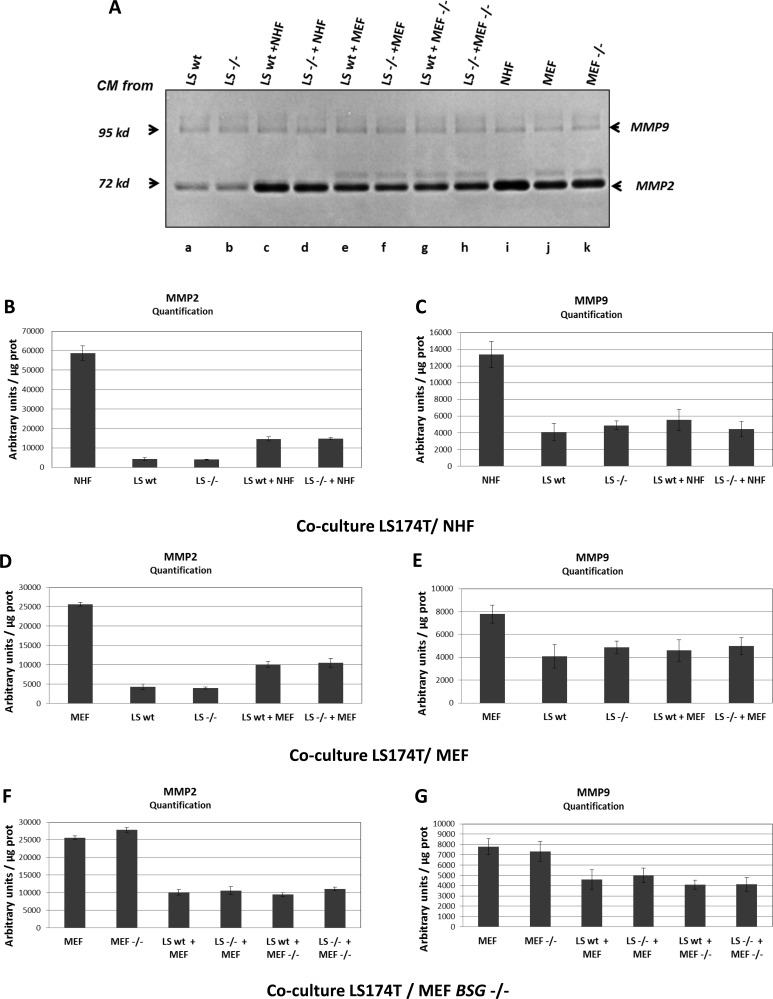
MMP2 and MMP9 expression in LS174T cells **A.** Zymogel analysis of MMP2/MMP9 activities in CM harvested after 48h of culture. LS174T wt or *BSG*^−/−^ (referred to as LS wt and LS−/−) cells were grown in 2% FBS media alone (a, b) or in co-culture with either NHF (c, d) or with MEF (wt or *BSG*^−/−^ cells refers to as MEF and MEF−/−) (e, f g, h). CM media of NHF, MEF wt and *BSG*^−/−^ cells cultured alone was also assessed (i, j, k) **B.** and **C.** Quantification of MMP2/MMP9 activities in CM of LS174T wt *vs BSG*^−/−^ cells cultured alone, or co-cultured with NHF. **D.** and **E.** Quantification of MMP2/MMP9 activities in CM of LS174T wt *vs BSG*^−/−^ cells cultured alone, or co-cultured with MEF wt. **F.** and **G.** Quantification of MMP2/MMP9 activities in CM of LS174T wt *vs BSG*^−/−^ cells cultured alone, or co-cultured with MEF *BSG*^−/−^. Results were normalized relative to protein quantity.

**Figure 3:**
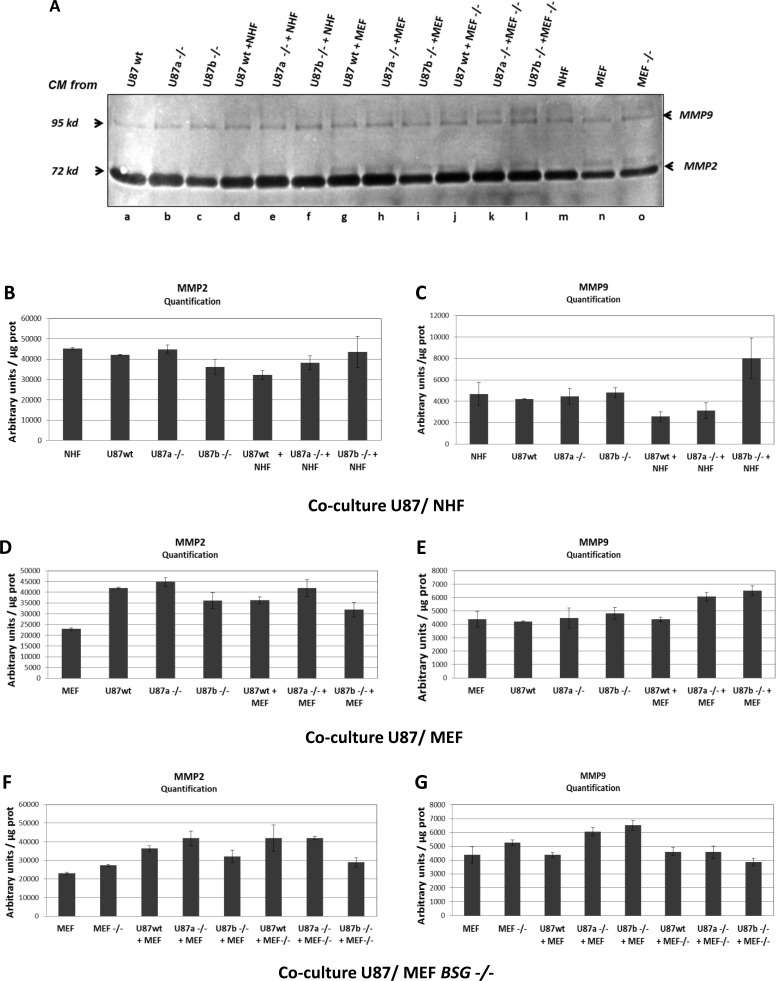
MMP2 and MMP9 expression in U87 cells **A.** Zymogel analysis of MMP2/MMP9 activities in CM harvested after 48h of culture. U87 wt or *BSG*^−/−^ cells (clones a, and b) (referred to as U87 wt and U87−/−) were grown in 2% FBS media alone (a, b, c) or in co-culture with either NHF (d, e, f) or with MEF (wt or *BSG*^−/−^ cells) (g, h, i, j, k, l). CM media of NHF, MEF wt and *BSG*^−/−^ cells cultured alone was also assessed (m, n, o) **B.** and **C.** Quantification of MMP2/MMP9 activities in CM of U87 wt *vs BSG*^−/−^ cells cultured alone, or co-cultured with NHF. **D.** and **E.** Quantification of MMP2/MMP9 activities in CM of U87 wt *vs BSG*^−/−^ cells cultured alone, or co-cultured with MEF wt. **F.** and **G.** Quantification of MMP2/MMP9 activities in CM of U87 wt *vs BSG*^−/−^ cells cultured alone, or co-cultured with MEF *BSG*^−/−^. Results were normalized relative to protein quantity.

As MMP2 and MMP9 are usually associated with tumour progression in a BSG-dependent way, we investigated their expression in a series of BASIGIN-positive human tumour cells. Zymogel analysis of MMP2 and MMP9 gelatinase activity showed that, while MMP9 is weakly expressed in all cells tested, MMP2 expression in extracellular medium varies between tumour cell lines. MDA-MB-231, LS174T and A549 cell lines express low levels of MMP2 (Figure 1C, lanes c, d, f and Figure 2A, lane a). In contrast, U87 cells express a significant level of MMP2, which is comparable to the expression by normal human fibroblasts (NHF) (Figure 1C lanes b, h and Figure 3A, lanes a, m) or mouse embryonic fibroblasts (MEF) (Figure 2A, lane j and Figure 3A, lane n). However the differential expression of MMP2 by tumour cells does not appear to reflect the expected metastatic properties of these cells since metastatic MDA-MB-231 cells express little or no MMP2 and MMP9 in the extracellular medium.

### Co-cultures of tumour cells with fibroblasts does not significantly change MMP2 and MMP9 expression in extracellular medium

We then tested in our models if, as published by [17], co-culture of tumour cells with fibroblasts was able to stimulate metalloproteases production in the extracellular medium. For this, we analysed MMP2 and MMP9 production in conditioned medium obtained from LS174T and U87 cultured alone, or with two different types of fibroblasts (NHF or MEF). Zymogels analysis, and quantification of MMP activities showed that co-cultures of LS174T with either NHF (Figure 2A, lane c and Figure 2B–2C) or MEF (Figure 2A, lane e and Figure 2D–2E) did not significantly produce more MMP2 and MMP9 than tumour cells or fibroblasts cultured alone (Figure 2A, lanes a, i, j and Figure 2B–2E). The same results were obtained with U87 cells as shown in Figure 3. Co-culture of U87 with either NHF (Figure 3A, lane d and Figure 3B–3C) or MEF (Figure 3A lane g and 3D–3E) did not produce more MMP2 and MMP9 than NHF or MEF alone (Figure 3A lanes a, m, n and 3B-3E). These results indicate that contrary to what has been previously published, fibroblasts and tumour cells do not seem to cooperate to produce more MMP in the extracellular medium *in vitro*. Conversely, it should be noted that NHF or MEF co-cultured with LS174T cells (but not with U87 cells) seem to produce less MMP2 and MMP9 than when they are cultured alone (Figure 2A lanes i, j compared to lanes c, d, e, f and Figure 2B–2E).

### Activation of NHF by conditioned media (CM) from tumour cells

**Figure 4:**
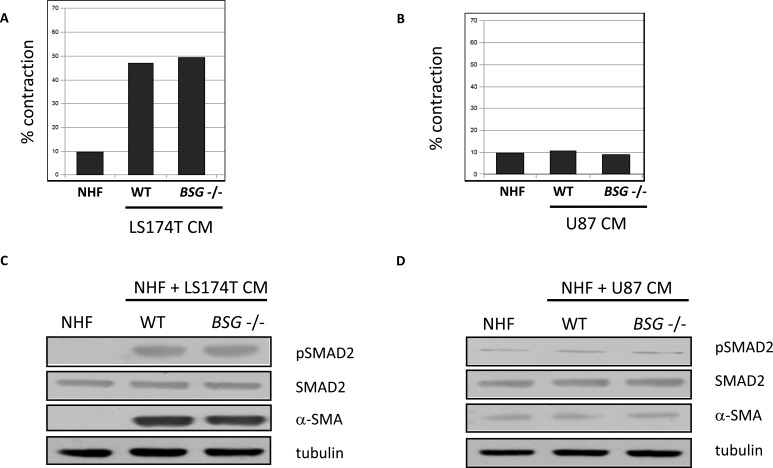
NHF activation by CM from tumour cells **A.** and **B.** Quantification of matrix contraction by NHF stimulated by CM from LS174T and U87 (wt *vs BSG*^−/−^) tumour cells. **C.** and **D.** Immuno-detection of pSMAD2 and α-SMA expressed in NHF 48h after stimulation by CM from LS174T and U87 (wt *vs BSG*^−/−^) tumours cells. SMAD2 and tubulin are used as control. These figures are representative of three independent experiments.

It is well known that when in contact with tumour cells, fibroblasts can acquire a specific CAF activated phenotype. This can result in the expression of new markers, such as alpha smooth muscle actin (α-SMA), the activation of TGFβ/SMAD signalling pathways and acquisition of new properties, such as matrix remodelling [24, 25]. We thus, tested if conditioned media from LS174T and U87 tumour cells are able to activate stimulated NHF *in vitro*. NHF stimulated by CM from LS174T cells seems to have acquired new matrix remodelling properties represented by their capacity to contract collagen gels (Figure 4A), while CM from U87 cells does not have any effect (Figure 4B). Moreover, immunoblotting analysis indicates that NHF stimulated by CM from LS174T cells express α-SMA, and that TGFβ/SMAD signalling pathways is activated in these stimulated fibroblasts (Figure 4C). On the contrary, CM from U87 cells does not have any effect (Figure 4D). These data suggest that fibroblasts stimulated with CM from LS174T cells could have acquired a status similar to that of activated CAFs. Nevertheless, BSG disruption had no impact on collagen contraction or on TGFβ/SMAD signalling pathway activation.

### Stimulation of fibroblasts by conditioned media from tumour cells does not significantly change MMP2 and MMP9 expression in extracellular medium

**Figure 5:**
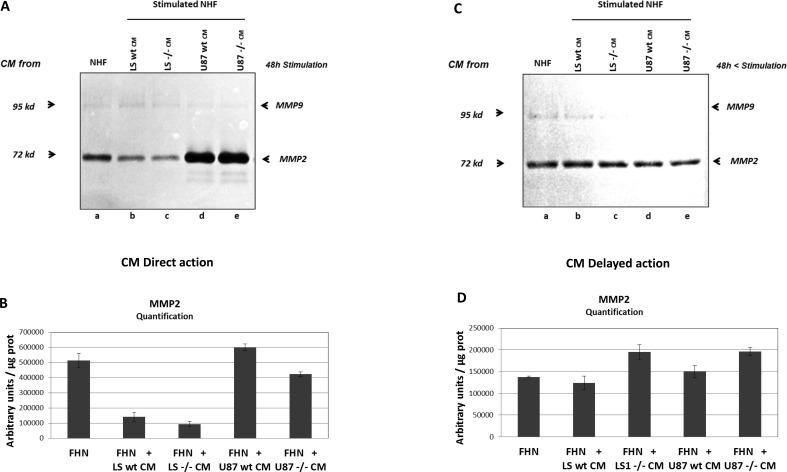
MMP2 and MMP9 expression by NHF stimulated by conditioned media (CM) from tumour cells **A.** Zymogel analysis of MMP2/MMP9 activities in NHF CM harvested after 48h of culture (CM direct action). NHF were grown for 48h in the presence of CM from NHF as a control (a), or from tumour cells, LS74T wt (b) *vs BSG*^−/−^ (c), U87 wt (d), *vs BSG*^−/−^ (e). **B.** Quantification of MMP2 activity in CM from stimulated NHF. **C.** Zymogel analysis of MMP2/MMP9 activities in NHF CM harvested after 48h of cell culture, washed and cultured in fresh 2% FBS media during an additional 48h (CM delayed action). **D.** Quantification of MMP2 activity in CM from NHF 48h after stimulation.

It is also known that activated fibroblasts are able to secrete increased level of matrix degrading proteases such as MMP2 and MMP9 [17, 28]. Therefore, we tested if CM from LS174T and U87 tumour cells could induce MMPs production in stimulated fibroblasts. Zymogel analysis and quantification of MMPs activity showed that conditioned media from LS174T or U87 cells have no significant effect on MMP2 production by stimulated NHF either by direct stimulation (Figure 5A lanes a, b, d and 5B), or by delayed action (Figure 5C, lanes a, b, d and 5D). In this experiment, due to the weakness of MMP9 signal, only MMP2 activity was measured. As in co-culture experiments, it should be noted that NHF directly stimulated by CM from LS174T (but not by CM from U87 cells) produce less MMP2 than non-stimulated NHF (Figure 5A lane a compared to lanes b, c d, e and Figure 5B).

### Genetic disruption of BASIGIN does not significantly affect MMP2 and MMP9 production by tumour and fibroblastic cells

The impact of BASIGIN on MMPs production by tumour cell lines was first evaluated by analysing the MMP2 and MMP9 activities in CM from wt *versus BSG*^−/−^ tumour cell lines alone, or co-cultured with fibroblasts. Zymogel quantification showed that BSG invalidation does not significantly affect MMP2 and MMP9 production by LS174T (Figure 1C lanes d, e, Figure 2A lanes a, b, and Figure 2B–2C), U87 (Figure 1C lanes h, i, Figure 3A lanes a, b, c and Figure 3B–3C), and A549 (Figure 1C lanes f, g) tumour cells.

MMP2 and MMP9 activities were also evaluated in MEF wt *versus bsg*^−/−^. We used MEF from BSG-null mice invalidated for BASIGIN by an insertion of a MC1neo box into the gene [30]. In these cells, as in ZFN invalidated cells, the disruption of *BSG* gene results in the total loss of the protein (data not shown). As observed in tumour cells, the invalidation of BASIGIN in MEF does not notably modify the level of MMP2 and MMP9 in extra cellular media (Figure 2A lanes j, k, Figure 3A lanes n, o and Figure 2F–2G, 3F-3G). Moreover, the invalidation of BASIGIN does not affect MMPs production when tumour cells were co-cultured with NHF (Figure 2A, lanes c, d, Figure 3A lanes d, e, f and Figure 2B–2C, 3B-3C), with MEF wt (Figure 2A lanes e, f, Figure 3A lanes g, h, i and Figure 2D–2E, 3D-3E) or MEF *bsg*^−/−^ (Figure 2A, lanes g, h, Figure 3A lanes j, k, l and Figure 2F–2G, 3F–3G). Finally, *BASIGIN* gene disruption does not have any detectable effect on the level of MMP2 and MMP9 produced by CM stimulated fibroblasts (Figure 5A and 5C lanes b, c, d, e and Figure 5B, 5D).

### Genetic disruption of BASIGIN does not significantly affect MMP2, MMP9, MMP3, MMP11 and MMP14 gene-expression in tumour cell lines

**Figure 6:**
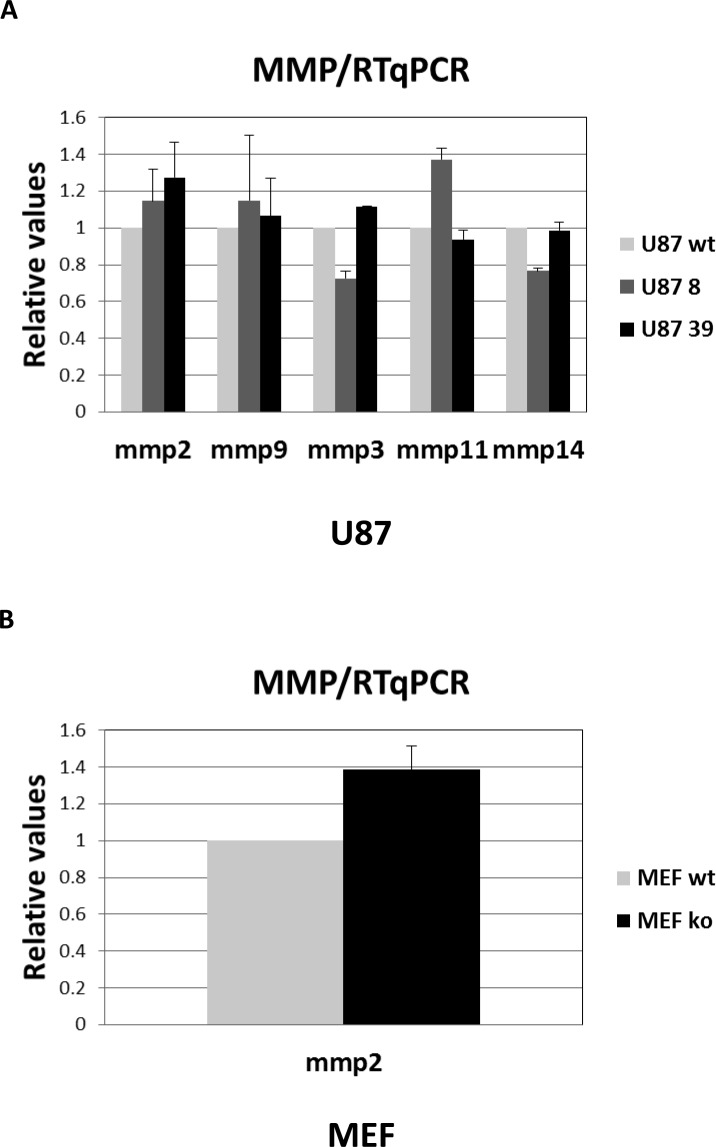
RT-qPCR analysis of MMP expression **A.** Quantification of relative MMP expression in U87 wt *vs BSG*^−/−^ cells. **B.** Quantification of relative MMP expression in MEF wt *vs BSG*^−/−^ cells.

In addition to MMP2 and MMP9, we investigated the expression of MMP3, MMP11, MMP13 and MMP14 in tumour cells. These MMPs have also been reported to be associated with cancer progression in a BSG-dependent manner. As these MMPs have no gelatinase activity, we could not evaluate their expression in the extracellular medium, but only at intracellular level. Due to high variability in the expression level of MMPs in tumour cells, and to standardize the results, we choose to use RT-qPCR method rather than immunodetection to measure the expression of these other MMPs. In confirmation of the zymogel results, RT-qPCR analysis showed that MMP2 and MMP9 mRNA are barely detectable in wt LS174T cells (CT>35), while wt U87 cells expressed a significant level of MMP2 and MMP9 mRNAs (Figure 6A). Concerning other MMPs, levels of MMP3, MMP11, MMP13 and MMP14 mRNA are too low in LS174T cells to be detected. In contrast, U87 cells expressed noticeable levels of MMP3, MMP11 and MMP14 as shown in Figure 6A. On the contrary to NHF, which express high level of all tested MMPs (data not shown), MEF do not express any of the tested MMPs, except MMP2 (Figure 6B). Nevertheless, the invalidation of the *BASIGIN* gene does not significantly affect MMP2, MMP9, MMP3, MMP11 and MMP14 expression at intracellular level in any of the cells tested (Figure 6A and 6B). It is to be noted that the MMP1 collagenase expression present in NHF (data not shown) was not detectable in tumour cells.

## DISCUSSION

In 1982, Biswas et al [17] showed for the first time that fibroblasts co-cultured with tumour cells, or treated by tumour cell conditioned media, produced an increased collagenase activity *via* a soluble factor present in tumour conditioned media. Then, they showed that this factor named tumour cell-derived collagenase stimulatory factor (TCSF) is present both as a soluble form and a tumour cell membrane component. Finally, in 1995 the same group identified TCSF as BASIGIN/CD147, renamed EMMPRIN for MMP inducer [31]. Since then, a large number of papers continue to be published establishing a correlation between BASIGIN and the tumorigenic capacities of cancer cells *via* MMPs induction.

### BASIGIN and the control of bioenergetics

However, in the meantime Halestrap's group demonstrated that BASIGIN is a unique accessory protein allowing the expression of MCT1 and MCT4 at the plasma membrane [6]. Several publications, including ours, clearly demonstrated that BASIGIN is involved in cancer cells through the direct functionality of MCT1 and MCT4. By assuring lactic acid export, BASIGIN indirectly contributes to intracellular pH changes [32–34], the rate of glycolysis and tumour growth [14–16]. Therefore, the question was raised whether the pro-tumour action of BASIGIN is linked to its role in glucose metabolism and bioenergetics, MMPs induction, or both. Because of the strong structural and physiological interdependent relationship between BASIGIN and MCT1/MCT4 for plasma membrane expression, it was difficult to uncouple the function and the expression of these three proteins. So to answer this question, we performed an experiment in which we pharmacologically and genetically ablated MCT1 and MCT4 functions respectively, keeping high plasma membrane expression of BASIGIN [14]. This experiment revealed that tumorigenicity declined with the loss of MCT function, independently of BASIGIN level. We recently confirmed these findings by showing that the knockout of BASIGIN alone in LS174 and U87 tumour cell lines re-programmed tumour cell bioenergetics from glycolysis to oxidative phosphorylation [16].

### BASIGIN/EMMPRIN and the control of Extracellullar Matrix Metalloproteases

In parallel, as reported extensively in the literature, the role of BASIGIN in the process of cellular invasion and metastasis *via* the induction of metalloproteases has remained very active, and with few exceptions, ignores the metabolic component. However, these publications are mostly based on indirect proofs since the experiments were often made using either BASIGIN overexpression, siRNA invalidation or forced expression with recombinant soluble BASIGIN [1, 18, 23, 28]. In this manuscript, we addressed the question of the direct involvement of BASIGIN in MMPs induction by exploiting four *BSG* knockout (KO) cell lines derived from human and mouse. First, using three human cancer cell lines, LS174, A549, and U87 (wt *versus BSG*^−/−^), we showed that *BSG* gene disruption did not significantly affect the MMPs production in extracellular media. The same results were obtained for intracellular expression of MMPs by using qPCR analysis. Then, we developed co-cultures experiments between fibroblasts and tumour cells, and measured MMPs production in the extracellular media. We showed that not only did BASIGIN disruption not affect MMPs production, but that co-cultures also did not significantly increase MMPs production in the media. This surprising finding indicates that, contrary to what has been previously reported, fibroblasts and tumour cells do not seem to cooperate to produce more MMPs in the extracellular medium. The use of mouse fibroblasts (MEF) lead to the same result, namely a lack of significant effects on MMPs production while comparing MEF-bsg^−/−^ *vs* MEF-wt, or in co-culture with tumour cells. How can we then explain these contradictory results? First of all as mentioned above, many previously published data are based on either forced expression (transfection or used of a purified recombinant soluble form of BASIGIN), or with incomplete invalidation (siRNA or antisense). Use of these methods showed therefore a correlation between BASIGIN and MMPs induction rather than a direct link. The results presented here stand out from previous reports because it is the first study conducted with *BASIGIN* KO where, in contrary to siRNA experiments, BASIGIN expression was totally abolished. In the same way, *BSG* KO performed in MEF by a neo box insertion and destruction of the *BASIGIN* gene function confirmed our results. Moreover, we pushed the study further by exploring the direct stimulation of fibroblasts with tumour cell-conditioned media as a more informative approach. Surprisingly, even under these conditions, where human fibroblasts acquired an activate profile quite similar to CAFs by presenting the α-SMA myofibroblastic marker, and activating TGFβ pathway, we failed to demonstrate a link between BASIGIN expression and MMPs. Therefore, we must conclude from this work that the direct link between BASIGIN, tumorigenicity and MMPs expression is not proven.

Thus if BASIGIN has a real effect on MMPs induction in cancer cells, this could be in an indirect way, and probably *via* its interaction with MCTs transporters. BASIGIN overexpression in tumour cells, that is usually associated with the overexpression of MCT1/MCT4, would be simply a reflection of a greater ability of the cells to export lactate and to grow in a hostile environment. The increased production of MMPs which accompany the tumour growth and the metastatic stage, would not be directly linked to the overexpression of BASIGIN, but more a consequence of the acquisition of aggressive capacities of the cells becoming best adapted to the acidic microenvironment by overexpressing MCTs.

Our findings complement and echo similar results obtained by Kolb's group [35] on mammary gland development. They showed that, contrary to what might be expected, BASIGIN has no direct effect on MMP gene expression during adult mammary gland development. Inactivation of *basigin* gene in mice [30] affected developmental processes that include defects in embryos implantation, arrested spermatogenesis, male and female infertility, abnormal behaviour deficits in vision and odour. However until now and despite many studies using BASIGIN-null mice, a direct role of MMPs and above all a direct link between basigin and MMP induction in these processes could not be clearly established. Furthermore, Philp's group demonstrated that vision deficits in *basigin*-null mice could be ascribed to the failure of MCTs in photoreceptor cells to properly locate in the plasma membrane [36]. Taken together these data reinforced our proposal that BASIGIN is involved in cancer progression through its MCTs chaperone function and not by MMP induction.

However, it remains to be explained by which mechanism soluble BASIGIN (from transfection or injection into the extra cellular media) manages to trigger MMPs production by treated cells, as it was reported in the literature. Many data from various studies indicate that the expression of MMPs is regulated at several levels, from gene transcription to activation, by multiple factors present in the extracellular matrix, such as TGFβ, EGFR, TNF-α, prostaglandin E2 and many others bioactive molecules. Moreover, depending on the cells, hypoxia and hyperglycaemia have been reported to regulate MMPs expression. More interestingly, these data showed that cell-extracellular matrix and cell–cell interactions bring an important contribution to the regulation of MMPs production at each level of regulation (for review [37]). In conclusion, the profile of MMPs production is cell specific, and the regulation of MMPs expression is a very complex process in which BASIGIN could play a minor role as a cell recognition molecule in a cell dependant manner. Indeed, we could imagine that, in a context of over-expression, BASIGIN, which is a very reactive cell surface protein due to its highly glycosylated structure, could have a cell recognition function, directly *via* cell-cell contact or indirectly *via* its soluble form and could activate MMPs production in a non-specific way *via* its contacts with the many extracellular matrix MMPs inducers.

At this stage we conclude that the induction of MMPs by BASIGIN as it has been initially hypothesized by Biswas [17, 31] is not proven and we propose to abandon the misleading acronym EMMPRIN.

## MATERIALS AND METHODS

### Cell culture

Normal Human Fibroblasts (NHF), Mouse Embryonic Fibroblasts (MEF), colon adenocarcinoma (LS174T), glioblastoma (U87), lung carcinoma (A549) and breast carcinoma (MDA-MB231) human cell lines were grown in Dulbecco's modified eagle medium (DMEM, Gibco) supplemented with 10% FBS, penicillin (10U/mL) and streptomycin (10μg/ml) except for conditioned media preparations where cells were cultured in 2% FBS.

### ZFN-mediated gene knockout of the BSG gene

LS174T, U87 and A549 BASIGIN expressing cell lines were transfected with the ZFN designed by Sigma-Aldrich (CKOZFN1227-1KT, CompoZr Custom ZFN) targeting exon 2 of *BSG* as previously described [16, 38]. Another LS174T *BSG* knockout cell line was generated with a ZFN targeting exon 7 of *BSG*, common to all spliced isoforms of the gene (Sigma-Aldrich, CSTZFN-1KT, CompoZr Custom ZFN). Transfected cells were detected with a CD147 (MAB972, R&D Systems) primary antibody and with a PE-conjugated anti-mouse IgG (115-115-164, Jackson ImmunoResearch) secondary antibody as described [14]. Several negative clones were detected by immunoblotting and only clones disrupted for the two alleles (DNA sequencing) were studied (cl18: *BSG*^−/−^ LS174T cells, cl8 and cl39: *BSG*^−/−^ U87 cells and cl153: *BSG*^−/−^A549 cells).

### Immunoblotting

Immuno-detection was performed as previously described [14]. Briefly, cells were lysed in 1.5X SDS buffer and protein concentration was determined by the BCA assay. Proteins (40 μg) were separated on 8% SDS polyacrylamide gels and transferred onto polyvinylidene difluoride membranes (Millipore). Blots were blocked in 5% milk in Tris-HCL/NaCl buffer and incubated with antibodies. Immunoreactive signals were revealed with horseradish peroxidase (HRP) antibodies (Promega) using ECL system (Amersham Biosciences). The antibody against BASIGIN (MAB972) was purchased from R&D systems, antibodies against SMAD2 (3122), pSer465/467-SMAD2 (3108), were purchased from Cell Signaling technology, and alpha-smooth muscle actin (Ab569) from Abcam. α-tubulin (T4026, sigma) and Arrest Defective-1 protein (ARD1) (produced in our laboratory [39]) were used as loading controls.

### Gelatin zymography (Zymogel) analysis

MMP activity was detected using zymogel technology [24]. Gelatin zymogels were performed as follows. After centrifugation, conditioned media samples from various cell lines were diluted (v/v) in loading buffer containing Tris-HCl pH 6.8, 2% SDS, 4% sucrose and bromophenol blue. Then, samples were separated by polyacrylamide gel electrophoresis (SDS free, 10% polyacrylamide gel containing 0.3% (w/v) gelatine). After electrophoresis, gels were first incubated in 2.5% triton for 90min, then in 50 mM Tris-HCl pH 7.5, 200mM NaCl, 2mM CaCl2, and 1mM MgCl2 buffer for 18h at 37°C. Gels were stained with Coomassie blue and washed in 10% acetic acid and 10% ethanol. MMP gelatinase activity was visualized by observing the light bands standing out against the dark background of the gel. The signal strength of these light bands was analyzed and quantified using the “gene tools” software from Syngene. Results were normalized to the amount of proteins.

### Conditioned media (CM) preparation

Cancer cells were grown to confluence, washed twice with PBS and then incubated in 2% serum media at 37°C. After 48h, CM was collected, centrifuged at 5000g for 5min to remove cell debris and the supernatant stored at −80°C. For immunoblotting analysis, fibroblasts were stimulated with cancer cells CM for 48h.

### Matrix remodelling assay

Matrix-remodelling assays were performed as previously described [40]. Fibroblasts were embedded (2.5×10^4^) in 100μl of matrix gel [41]. After 1h at 37°C, matrices were overlaid with 100μl of CM from different conditions. The CM was changed every 2 days and at day 6 gel diameters were measured using image J. The contraction of the gel was calculated using the formula: 100x(well diameter-gel diameter)/well diameter.

### RT-qPCR analysis

Total RNA was extracted from various cell lines using the RNA extraction kit from Qiagen according to the manufacturer's instructions. cDNA synthesis was performed by the Quantitect reverse transcription kit (Qiagen). The relative expression of various MMPs and BSG mRNA was determined by real-time quantitative PCR (qPCR) using TaqMan nucleotide primers probes (Life Technologies, references will be provided upon request) and qPCR master mix buffer (Eurogentec). Real-time qPCR experiments were performed on a “step one plus” system.

### Statistical analysis

Data are expressed as mean +/− SEM. Each experiment was performed at least three times and the number of experiments is represented by n. statistical analysis was done with the unpaired student's test. Differences between groups were considered statistically significant when *p* < 0.05.
